# An Unusual and Rare Presentation of Dermatomyositis Sine Dermatitis Complicated by Neuromyositis

**DOI:** 10.7759/cureus.10000

**Published:** 2020-08-24

**Authors:** Sami Rabah, Cristabel Robles Hidalgo, David Sternman, Clare Bryce

**Affiliations:** 1 Internal Medicine, Lincoln Medical Center, Bronx, USA; 2 Neurology, Lincoln Medical Center, Bronx, USA; 3 Pathology, Mount Sinai Hospital, New York, USA

**Keywords:** dermatomyositis, myopathy, neuromyositis, weakness, muscle biopsy, autoimmune

## Abstract

Dermatomyositis sine dermatitis (DMSD) is a rare autoimmune disease. It's distinguished from classical dermatomyositis (DM) by a lack of skin involvement. DM is known to have a variety of extramuscular manifestations, including interstitial lung disease, myocarditis, and dysphagia. However, peripheral nervous system involvement in DM termed "Neuromyositis" is less often encountered. Neuromyositis diagnosis is controversial due to its rarity, unknown mechanism, and heterogeneity of nerve pathology findings. A 53-year-old woman presented to our hospital following a fall. Six months prior to presentation, she had a sensory disturbance in her right foot. On admission, she had a right foot drop that progressed to right then left lower extremity weakness. A biopsy of the superficial peroneal nerve and long peroneal muscle showed large fiber nerve axonal loss, CD20 B-cell and CD4 T-cell predominant inflammatory infiltrate involving the perimysial connective tissue of the muscle, as well as myocyte hypertrophy and fibrosis with type I fiber predominance. These findings were compatible with dermatomyositis with neuropathic features. Electrophysiological studies of lower extremities revealed severe widespread axonal dysfunction, as evidenced by decreased tibial compound muscle action potential (CMAP), no peroneal motor responses, absent sural sensory nerve action potential (SNAP), and extensive active denervation throughout the left lower extremity. Three months later, she developed bilateral upper extremity weakness. A biopsy of the deltoid muscle that was done eight months after admission showed CD20 B-cell and CD4 T-cell predominant inflammatory infiltrates involving the perimysial connective tissue. These findings were pathologically similar to the first biopsy. Subsequently, a repeat electromyography (EMG) of upper extremities revealed myopathic changes with normal nerve conductions. She ultimately became quadriplegic and ventilator-dependent nine months after admission. She never exhibited any skin findings throughout her course of illness. An extensive imaging and laboratory workup did not reveal any occult malignancy, inflammation, or nutritional deficiency. Our patient did not respond to steroids or intravenous immunoglobulin (IVIg) and ultimately passed away. The clinical, pathological, and electrophysiological features suggested the presence of neuromyositis. This case illustrates the importance of recognizing peripheral nervous system involvement as a significant and yet underreported extramuscular manifestation of DM. There are currently no formal management guidelines for neuromyositis.

## Introduction

Dermatomyositis is an autoimmune idiopathic inflammatory myopathy. It’s characterized by symmetric proximal muscle involvement and is known to have multiple cutaneous findings such as Gottron’s papules, Gottron’s sign, and heliotrope rash [1]. Dermatomyositis sine dermatitis (DMSD) is distinguished from classical dermatomyositis (DM) by a lack of skin findings [1]. DM is known to have a variety of extramuscular manifestations, including dysphagia, heart failure, and interstitial lung disease [1]. However, peripheral nervous system involvement in DM termed “Neuromyositis” is rare [2]. The term “Neuromyositis” was introduced by Senator in 1893 to describe the concomitant involvement of the peripheral nervous system in DM [3]. Since then, very few cases of neuromyositis have been reported in the United States, Europe, China, Japan, and Korea [2]. The mechanism and pathology behind this disease are still unclear [4]. Here, we report an unusual case of DMSD associated with progressive severe axonal peripheral neuropathy unresponsive to treatment.

## Case presentation

A 53-year-old African-American female with a past medical history of morbid obesity, hypertension, bipolar disorder, anxiety, and asthma, presented to the hospital with right lower extremity weakness, numbness, and right foot drop. She had multiple falls in the past few weeks before the initial presentation and was complaining of right leg numbness and urinary incontinence for the past six months. Cervical magnetic resonance imaging (MRI) revealed compressive cervical myelopathy due to disc protrusions. She subsequently underwent a corpectomy of C6 and an anterior cervical discectomy and fusion (ACDF) of C4-C7 and was transferred to a rehabilitation facility. Two months later, she was readmitted after developing complete loss of strength in her right lower extremity (LE) that had progressed to involve her left lower extremity. Physical exam at presentation revealed complete loss of strength and sensations in her right lower extremity, Left lower extremity’s strength exam revealed: hip flexion 0/5, hip abduction and adduction 2/5, quadriceps 4/5, hamstring 3/5, tibialis anterior 4/5, gastrocnemius 4/5. Deep tendon reflexes were diminished in both lower extremities, and no rash was observed at the time.

To diagnose the etiology of her weakness, we performed a series of tests. Laboratory investigations (Table 1) showed a mildly elevated creatinine kinase (CK), aldolase, C-reactive protein concentration (CRP), and erythrocyte sedimentation rate (ESR). Various autoantibodies tested were not detected. Rheumatoid factor and antinuclear-antibodies titers were also not elevated. She had normal protein electrophoresis. Viral serological tests for hepatitis B and C were negative, and a lumbar puncture (LP) showed normal cerebrospinal fluid (CSF) analysis. A lumbosacral MRI showed no nerve root compression. However, it revealed symmetric areas of edema within the paraspinal and pelvic musculature that was consistent with myositis. Other imaging studies that included computed tomography (CT) of chest, abdomen, and pelvis did not reveal any abnormalities suggestive of a malignancy.

**Table 1:** Laboratory Data P-ANCA: perinuclear antineutrophil cytoplasmic antibodies, C-ANCA: cytoplasmic antineutrophil cytoplasmic antibodies, Anti-MAG: anti-myelin-associated glycoprotein, Anti-JO-1: anti-histidyl-tRNA synthetase, Anti-PL-7: anti-threonyl-tRNA synthetase, Anti-PL-12: anti-alanyl-tRNA synthetase, Anti-OJ: anti-glycyl-tRNA synthetase, Anti-EJ: anti-isoleucyl-tRNA synthetase, Anti-SRP: anti-signal recognition particle, Anti-MI-2: Helicase protein of nucleosome remodeling deacetylase complex antibodies, Anti-TIF-gamma: anti-transcriptional intermediary factor-1 gamma, Anti-MDA-5: anti-melanoma differentiation-associated gene 5, Anti-PM/SCl-100: polymyositis and scleroderma, RNP: ribonucleoprotein. Anti-KU: DNA binding protein antibodies, Anti-SS-A: Sjögren's-syndrome-related antigen A.

Variable	Reference range, Adults	Results
Hemoglobin (g/dl)	12-16	12
White cell-count (10⁹/L)	4.8-10.8	7.1
Platelets count (10⁹/L)	150-400	252
Prothrombin time INR (sec)	0.9-1.34	0.98
Activated partial thromboplastin time (sec)	25-35.3	29.4
D-dimer (ng/ml)	<499	395
Alanine aminotransferase (U/L	13-56	13
Aspartate aminotransferase (U/L)	15-37	11
Creatine kinase total (U/L)	26-192	405
Aldolase (U/L)	3.3-10.3	11.4
C-reactive protein (mg/dl)	0-0.4	0.95
Erythrocyte sedimentation rate (mm/hr)	0-20	30
P-ANCA (units)	<20 units	<5 units
C-ANCA (units)	<20 units	<5 units
Anti-Nuclear Antibodies Titers		<1:80
Complement Levels		
C3 (mg/dl)	81-157	172
C4 (mg/dl)	13-39	60
Anti-MAG antibody	Negative	Negative
Myositis Antibodies Panel		
Anti-JO-1	<20 units	<20 units
Anti-PL-7	Negative	Negative
Anti-PL-12	Negative	Negative
Anti-EJ	Negative	Negative
Anti-OJ	Negative	Negative
Anti-SRP	Negative	Negative
Anti-MI-2	Negative	Negative
Anti-TIF-gamma	<20 units	<20 units
Anti-MDA-5	<20 units	<20 units
Anti-PM/SCl-100	<20 units	<20 units
U3 RNP	Negative	Negative
U2 snRNP	Negative	Negative
Anti-U1-RNP	<20 units	<20 units
Anti-KU	Negative	Negative
Anti-SS-A	<20 units	59

**Figure 1:**
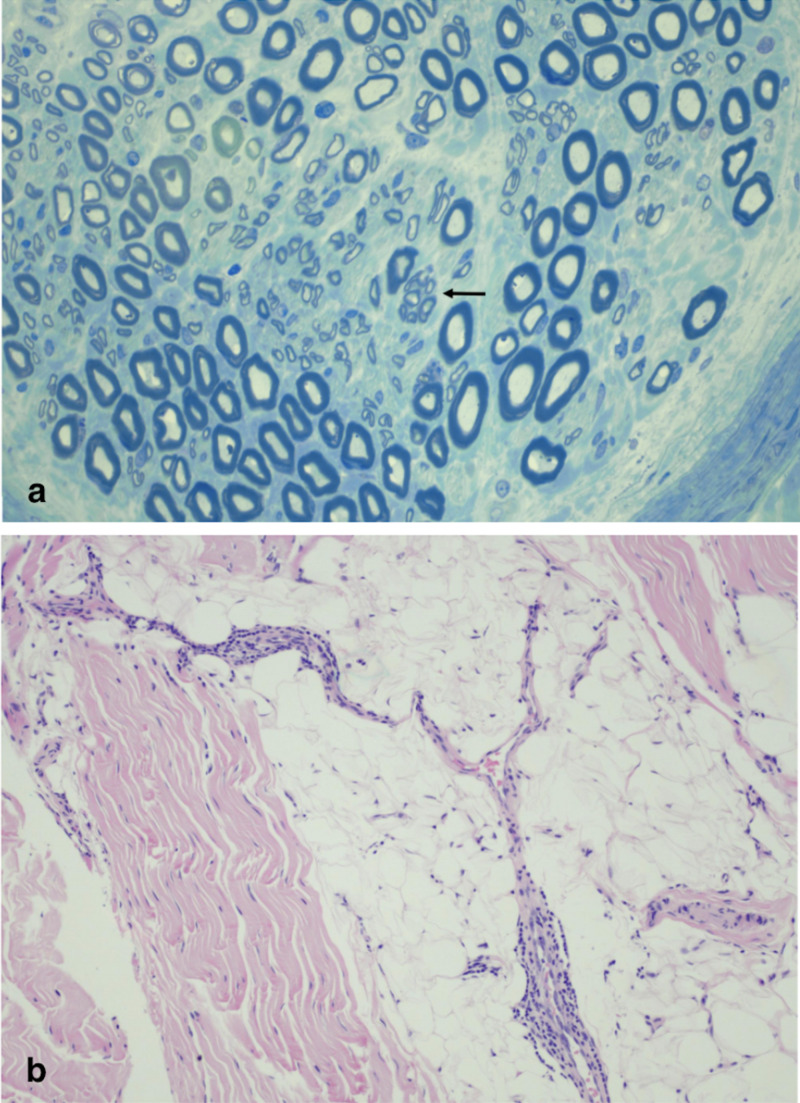
Biopsy of Superficial Peroneal Nerve and Long Peroneal Muscle Figure 1a: Peripheral nerve with focal depletion of myelinated axons and axonal regeneration clusters suggestive of axonal neuropathy. Arrow indicates a regeneration cluster (Epon section stained with toluidine blue). Figure 1b: Perimysial connective tissue inflammation can be seen, predominantly perivascular (hematoxylin and eosin (H&E)).

Initial nerve conduction studies (NCS) of LE (Table 2) showed profoundly diminished tibial nerve compound muscle action potential (CMAP) amplitude and no peroneal responses distally or proximally with absent sural and superficial peroneal sensory responses. Electromyography (EMG) of LE musculature (Table 3) revealed signs of extensive axonal denervation. In light of these findings, a biopsy of the superficial peroneal nerve and long peroneal muscle was done. Pathology findings showed mixed myopathic and neuropathic features, myocyte hypertrophy with a dominance of type I fibers, CD20 B-cell and CD4 T-cell predominant chronic inflammatory infiltrate involving the perimysial connective tissue, and presence of nuclear bag fibers and atrophic angular fibers without fiber type grouping which is consistent with acute denervation (Figure 1).

**Table 2:** Nerve Conduction Study (NCS) of Lower Extremities Lat: latency, Amp: amplitude, CV: conduction velocity.

Motor Nerve	Lat (ms)	Amp (mV)	CV (m/s)
Left Common Peroneal	0	0	0
Left Tibial	13.4	0.7	42.5
Sensory Nerve			
Left Sural	0	0	0
Left Superior Peroneal	0	0	0

**Table 3:** Electromyography (EMG) of Lower Extremities MUAP: Motor unit action potential, IA: insertional activity, Fib: fibrillation potentials, PSW: positive sharp waves, Fasc: fasciculation, Amp: amplitude, Dur: duration, PPP: polyphasic potential

Muscle	Spontaneous				MUAP			Recruitment	
	IA	Fib	PSW	Fasc	Amp	Dur	PPP	Pattern	Firing rate
Left Tib Anterior	Increased	3+	3+	None	Decreased	Increased	None	Discrete	Increased
Left Gastrconemius (Med)	Increased	3+	3+	None	Decreased	Increased	None	Discrete	Increased

Three months after admission, the weakness had spread to involve both left and right upper extremities (UE). Physical exam of muscle strength in upper extremities revealed: shoulder abduction 4/5, arms extension 4/5, digit extension 5-/5. Her left hand was mildly numb to touch. By then, there was a complete loss of strength in bilateral LEs. The patient was started on a course of corticosteroids but with little to no improvement. A subsequent biopsy from the deltoid muscle done eight months after admission showed CD20 B-cell and CD4 T-cell predominant inflammatory infiltrate involving the perimysial connective tissue, scattered regenerating and degenerating fibers, increased perimysial and endomysial fibrosis, type I fiber predominance, and myophagocytosis. No rimmed vacuoles were seen with the Gomori trichrome stain. These pathological findings were consistent with a predominantly myopathic process. EMG of right UE muscles (Table 4) showed signs of irritable advanced myopathy with proximal predominance. She was later started on azathioprine but had no improvement. 

**Table 4:** Electromyography (EMG) of Upper Extremities MUAP: Motor unit action potential, IA: insertional activity, Fib: fibrillation potentials, PSW: positive sharp waves, Fasc: fasciculation, Amp: amplitude, Dur: duration, PPP: polyphasic potential

Muscle	Spontaneous				MUAP			Recruitment	
	IA	Fib	PSW	Fasc	Amp	Dur	PPP	Pattern	Firing rate
Right Deltoid	Increased	1+	1+	None	Decrease	Increased	Present	Discrete	N
Right Triceps	Increased	1+	1+	None	Decrease	Increased	Present	Low mixed	N
Right Biceps	N	None	None	None	Decrease	Increased	Present	High mixed	N

Seven months after admission, the patient developed a weak cough, dysphagia, and her phonation was decreased. Weakness eventually spread to involve her respiratory muscles, which caused her to become ventilator-dependent. She received two courses of intravenous immunoglobulin (IVIG) and was finally maintained on methotrexate. This medication regimen failed to alter the course of her progressive weakness. The patient ultimately had a cardiopulmonary arrest and passed away.

## Discussion

Dermatomyositis is a multisystemic disorder characterized by progressive inflammatory myopathy and dermatitis. It is known to have a variety of extramuscular manifestations, including lung and cardiac diseases [1]. Patients with DM can develop chronic respiratory failure from interstitial lung disease and less commonly from respiratory muscle weakness [5].

DM is classified into different entities based on skin and muscle involvement. Dermatomyositis sine dermatitis (DMSD) is diagnosed when certain diagnostic criteria are met, and these include symmetric proximal muscle weakness, elevation of CK, EMG findings suggestive of myopathy, muscle biopsy with characteristic DM pathological findings, and absence of skin manifestations such as Gottron’s papules and heliotrope rash [1]. In one study, DMSD was found to have a prevalence of 8% in patients with a biopsy-proven DM [6]. The adermopathic period required for the diagnosis of DMSD is unknown, but diagnosis can be made based on the absence of a skin rash at the time of muscle biopsy [6]. Our patient didn’t have any rash throughout the course of her illness and had mildly elevated CK. Only 70-80% of patients with DM have elevated CK [7]. 

Our patient had evidence of muscle involvement, as shown by muscle biopsies. She also had concomitant peripheral neuropathy that was evident clinically and confirmed pathologically based on the superficial peroneal nerve biopsy findings, and electrophysiologically by the NCS findings of diminished CMAP amplitude of the tibial nerve. Peripheral nervous system involvement in DM termed “Neuromyositis” poses a diagnostic challenge for clinicians, as this entity is controversial due to its rarity, unknown mechanism, and heterogeneity of nerve pathology findings across previous case reports [2,4]. Although the pathognomonic mechanism of neuromyositis is unknown, some studies suggested that a vasculitic process induced by overproduction of vascular endothelial growth factor (VEGF) is involved while others suggested the presence of capillary endothelial ischemia [2,4]. 

We conducted an extensive workup to rule out other possible causes of neuropathies and found no evidence of vitamin deficiencies, infections, or malignancies. We have also considered the presence of other neurological pathologies, such as amyotrophic lateral sclerosis (ALS). However, ALS doesn’t cause sensory deficits and usually presents with upper motor neuron signs [8]. The patient didn’t meet the criteria for definitive DM. The subsequent deltoid biopsy showed perimysial connective tissue inflammation in addition to increased perimysial and endomysial fibrosis; this was consistent with the EMG findings of irritable advanced myopathy. In light of these findings, the possibility that the same pathological process affecting both muscle and nerve should be considered. 

There was no clinical improvement following a variety of medications, which included steroids, azathioprine, methotrexate, and intravenous immunoglobulin (IVIG). As of today, there are no specific therapies or management guidelines for neuromyositis. As previous case reports showed a varying response to steroids and other immunosuppressants [2], more clinical studies are needed to guide clinicians on treatment.

## Conclusions

We have described an unusual presentation of DM. Our patient presented with dermatomyositis sine dermatitis (DMSD) that was complicated by the presence of neuromyositis which was diagnosed clinically, pathologically, and electrophysiologically. An extensive workup didn’t reveal any other underlying disease that could have explained the presence of peripheral neuropathy. The association between neuromyositits and dermatomyositis is a diagnostic challenge for physicians and is considered controversial due to its rarity, unknown mechanism, and heterogeneity of nerve pathology findings. There are no well-designed studies on neuromyositis. Further studies are needed to explore neurological involvement in DM to guide diagnosis and treatment.
